# Feasibility study of personalized speed adaptation method based on mental state for teleoperated robots

**DOI:** 10.3389/fnins.2022.976437

**Published:** 2022-09-02

**Authors:** Teng Zhang, Xiaodong Zhang, Zhufeng Lu, Yi Zhang, Zhiming Jiang, Yingjie Zhang

**Affiliations:** ^1^School of Mechanical Engineering, Xi’an Jiaotong University, Xi’an, China; ^2^Shaanxi Key Laboratory of Intelligent Robot, Xi’an Jiaotong University, Xi’an, China

**Keywords:** personalized, teleoperated robots, biosignals, reinforcement learning, mental state, EEG and EOG

## Abstract

The teleoperated robotic system can support humans to complete tasks in high-risk, high-precision and difficult special environments. Because this kind of special working environment is easy to cause stress, high mental workload, fatigue and other mental states of the operator, which will reduce the quality of operation and even cause safety accidents, so the mental state of the people in this system has received extensive attention. However, the existence of individual differences and mental state diversity is often ignored, so that most of the existing adjustment strategy is out of a match between mental state and adaptive decision, which cannot effectively improve operational quality and safety. Therefore, a personalized speed adaptation (PSA) method based on policy gradient reinforcement learning was proposed in this paper. It can use electroencephalogram and electro-oculogram to accurately perceive the operator’s mental state, and adjust the speed of the robot individually according to the mental state of different operators, in order to perform teleoperation tasks efficiently and safely. The experimental results showed that the PSA method learns the mapping between the mental state and the robot’s speed regulation action by means of rewards and punishments, and can adjust the speed of the robot individually according to the mental state of different operators, thereby improving the operating quality of the system. And the feasibility and superiority of this method were proved. It is worth noting that the PSA method was validated on 6 real subjects rather than a simulation model. To the best of our knowledge, the PSA method is the first implementation of online reinforcement learning control of teleoperated robots involving human subjects.

## Introduction

As a branch of the field of robotics, teleoperated robotic systems have received extensive attention from academia and industry due to their advantages such as remote operation and operation in hazardous environments. In this system, the operator guides the robot movement to perform the task. In the process of the robot performing a task, the operator, as a part of the system, can learn the task execution status through information perception or feedback, and can also control the robot by sending commands. Teleoperated robotic systems are mostly used in special operations such as deep-sea exploration, space operations, detoxification and detonation and precision surgery (Nuño et al., 2011; Zhai and Xia, 2016). Due to the characteristics of high risk, high precision and high difficulty in this field, the amount of information that operators need to process has increased dramatically, resulting in higher mental workload and mental pressure. Studies have shown that higher mental workload and mental pressure will cause rapid mental fatigue, decreased vigilance, stress reaction, and increase people’s errors and frustration (Zhang T. et al., 2019). On the contrary, too low mental workload and mental pressure will cause a waste of human resources or cause disgust. These adverse mental states will lead to errors in information acquisition, analysis, and decision-making, which will further lead to the decline of job performance and even lead to safety problems caused by human accidents (Wilson, 2005; Catelani et al., 2021).

To overcome this problem, various adjustment methods have been proposed. As early as in the 1990s, an adaptive automation method was first proposed. Parasuraman (1993), Scerbo et al. (2003), Kaber et al. (2016) scholars published a series of papers to discuss adaptive automation concept and theoretical issues. It is defined as an automated human-computer interaction system design method that can change the level of automation by users and systems. This method can dynamically adjust the automation level or working mode at any time according to the operator’s mental state to match the operator’s needs and mental state (such as the mental workload state, fatigue state, etc.), thus achieving the purpose of improving the operation performance and reducing human errors (Parasuraman, 1993; Scerbo et al., 2003; Kaber et al., 2016).

In recent years, adjustment methods based on physiological signal detection has been widely accepted with its unique advantages. Firstly, neurophysiological measures could be obtained continuously and online. Secondly, the neurophysiological ones may be recorded continuously without using overt responses (i.e., additional tasks) and may provide a direct measure of the operator’s mental (covert) activities. Also, neurophysiological measures have good resolution and form a good complementarity with performance measures (Di Flumeri et al., 2015). Finally, neurophysiological measures can be used not only to trigger the adjustment system but also to highlight why adjustment method is important for enhancing safety in high-risk and high-demanding tasks (Arico et al., 2016). From the late 1990s to the recent years, most of the studies have proved the positive effect of adjustment method in improving the system performance and subjective feeling using electroencephalogram (EEG), functional near-infrared spectroscopy (*fNIRS*), electro-oculogram (EOG), and heart rate variability (HRV) physiological parameters (Freeman et al., 1999; Di Flumeri et al., 2019; Li et al., 2019; Zhang X. et al., 2019; Wu E. Q. et al., 2021).

Among all these studies, EEG-based adjustment method is getting much attention. Parasuraman and Wilson (2008) proposed an adjustment system based on EEG detection, which can realize real-time detection of the operator’s mental state and dynamically assign task attributes and levels between machine and human according to mental state, thus effectively improving task performance. Jia et al. (2014) proposed an adjustment system for teleoperated robot tasks, which can detect the mental state according to the EEG of the operator and adjust the running parameters of the robot in real-time according to the mental state. The robot’s speed is increased and the response time is decreased when the operator is in an excellent mental state; on the contrary, when the operator is in a bad mental state, the robot’s speed is reduced and the response time is improved. Therefore, the adjustment system effectively improves control accuracy and security (Jia et al., 2014). Yang and Zhang (2013) used the fuzzy modeling method to establish an operator mental state estimation and prediction model based on EEG. Once the operator is found to be in a high-risk mental state, the model will immediately adjust its task load or remind the operator to take some measures to make the operator’s task match with its current mental state (Yang and Zhang, 2013). Pietro Arico used the passive brain-computer interface technology to detect the operator’s mental workload in the realistic air traffic control environment and took it as the indicator to trigger the adjustment system. Meanwhile, the technology’s effectiveness was verified in the realistic air traffic management system (Arico et al., 2016).

Although adjustment technology, especially EEG detection-based adjustment method, has achieved remarkable results, it is undeniable that there are still challenges to be solved. Firstly, due to the existence of individual differences, the relationship between mental state and operational quality is also different (Jia et al., 2012), and as the user’s operational skills improve, the relationship between mental state and operational quality will also change. This puts the conventional method based on static and fixed adjustment strategies into a predicament, because it does not have the ability of individual adjustment, and at the same time, it cannot change the adjustment strategy with the improvement of the operator’s skills. Secondly, most studies predetermine the “good or bad” characteristics of mental states, therefore, the task becomes more complicated when the “good” mental state is detected and less complicated when the “bad” mental state is detected. What makes this subjectivity wrong is the ignorance of mental states’ diversity, which is particularly strong across individuals and across time. Also, because the brain is in a highly dynamic and non-linear environment, mental states and behaviors are not one-to-one correspondence, but many-to-one, one-to-many or mixed cross correspondence (Abbass et al., 2014). As a result, most of the existing adjustment strategy is out of a match between mental state and adaptive decision, which cannot effectively improve operational quality and safety. Thirdly, the stability and robustness of adjustment systems based on EEG detection alone need to be improved, and adjustment systems for the fusion of multiple bioelectrical signals are the trend of development (Laurent et al., 2013; Wu W. et al., 2021).

To address these three problems, by introducing the idea of policy gradient reinforcement learning (Mnih et al., 2015) and combining the advantages of EEG and EOG, a personalized speed adaptation (PSA) method was proposed. Then, its feasibility was verified by designing a teleoperation experiment. Prominently, The PSA method belongs to a “human-in-the-loop” reinforcement learning framework, it is an interactive learning technology, which uses the interaction between the agent and the environment, and records each reward and punishment as personalized feedback to update the adjustment strategy. Compared with the methods based on static and fixed adjustment strategies, it has better dynamic adaptability. Secondly, reinforcement learning problems are usually normalized as Markov decision process (MDP), so this makes the PSA model have the natural characteristics of modeling mapping sequences (Arulkumaran et al., 2017), which can fully characterize the sequence features and capture the individual characteristics of operators. Moreover, the setting of the exploration mechanism can make the agent more fully explore the state and action space, which improves the diversity of results to a certain extent. Thirdly, since this type of model often aims to maximize the cumulative reward of the system, that is, the long-term feedback of the user’s operational quality is the optimization goal to update the adjustment strategy, so it can adapt to the development trend of the operator’s personalization. Finally, for a “human-in-the-loop” reinforcement learning training process, it’s arguably better for the algorithm to learn certain repetitive subsequences of actions (or patterns of actions) and store them in a rule-based fashion. Once an action pattern has been shown to be successful in multiple instances of a task context, it can be applied in similar other task contexts. This reinforcement learning convergence strategy can cope well with dynamic task environments (Wen et al., 2020; van Zoelen et al., 2021). It is worth noting that the mental state in this article does not refer to a specific discrete state, but a continuous state, which is mainly evaluated by the two indicators of arousal and valence in the dimension theory of psychology (Russell, 2003). The arousal represents the neurophysiological activation level of the subject, and the lower the degree of arousal, the stronger the degree of fatigue in the mental state, and vice versa. The valence indicates the positive or negative of the subject’s emotional state, and the lower the degree of valence, the stronger the negative degree in the mental state, and vice versa. Changes in the operator’s mental state in these two dimensions (indicators) will lead to changes in the quality of the operation. For example, as the degree of arousal decreases, that is, the degree of fatigue of the operator increases, the quality of the operation will be degraded or even human error will occur (Chuang et al., 2018). As the valence decreases, that is, the degree of negative mental state of the operator increases, the quality of the operation decreases (Jin et al., 2017). Moreover, the advantage of this setting using continuous indicators to evaluate mental state is that the influence of the diversity of mental states on operational quality can be fully considered. In addition, the PSA method has the following three advantages: (1) The PSA method is an end-to-end learning method that learns the mapping between mental states and robot speed regulation instructions through rewards and punishments, and does not need to explicitly identify which specific mental state it is, thus overcoming the above challenge 2. (2) The PSA method is individually trained for each operator, and with the increase of usage time, each operator’s personalized interaction habits, skill growth, etc., will optimize the PSA model parameters, thereby overcoming the above challenges 1. (3) The PSA method utilizes multimodal bioelectrical signals combining EEG and EOG in mental state perception, and fuses them on the feature layer, thereby overcoming the above challenges 3.

The major contributions of the paper can be summarized in three aspects. Firstly, according to the characteristics of the tele-robot system and the advantages of human and computer, a dual-loop human- machine information solution interaction mechanism was designed. By introducing the idea of policy gradient reinforcement learning, a mental state-based PSA model was constructed. Secondly, the PSA algorithm was developed which includes three steps, which are multimodal bioelectrical signal data preprocessing, mental state feature extraction and model efficient training. Finally, due to the high cost of data acquisition and labor-intensive problems in the “human-in-the-loop” reinforcement learning method (Nielsen et al., 2015; Alamdari et al., 2020), this paper collects a large number of experimental data with real human participation by designing two experimental paradigms of teleoperation robots with engineering value. The data not only proves the effectiveness of the PSA method, but also provides valuable knowledge and experience for future adjustment system design. The remaining of this paper is organized as follows. Section “Methodologies” describes PSA models and methods. Section “Materials and experiments” presents experimental materials, experimental paradigms, and data processing procedures. The results are presented in Section “Results.” Remarks and discussions are presented in Section “Discussion,” followed by the conclusion in Section “Conclusion.”

## Methodologies

We first designed a dual-loop human-computer information interaction mechanism according to the characteristics of the teleoperated robot system and the respective advantages of humans and computers. Secondly, by introducing the idea of policy gradient reinforcement learning, the PSA framework that could individually adjust the speed of the robot according to the mental state of different operators was constructed. Thirdly, the PSA problem was formulated and analyzed, and a mathematical model was established. Finally, the convergence criteria of the PSA model were defined.

### Personalized speed adaptation framework

**FIGURE 1:**
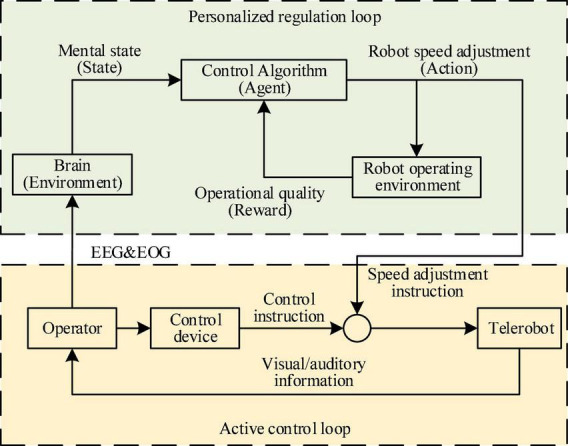
The figure shows the overall framework of the personalized speed adaptation (PSA) method.

The PSA method proposes a dual-loop human machine information interaction mechanism composed of the active control loop and the personalized regulation loop, as shown in Figure 1. In the active control loop, the operator sent control instruction to the robot through the control device, and supervised the running state of the robot through visual and auditory information, thus adjusting control instruction in real-time, and correcting sudden errors. In order to solve the problem that the operator’s mental state leaded to poor operational quality or even danger, a personalized regulation loop was designed on the basis of the active control loop. By introducing the idea of policy gradient reinforcement learning, the operator’s brain was innovatively used as the environmental element, the control algorithm (CA) as the agent element, the mental state as the state element, the speed adjustment instruction as the action element, and the operational quality as the reward element. Then, an end-to-end PSA model was established, which took as input the multimodal bioelectrical signals composed of EEG and EOG that reflect the operator’s mental state, and used the robot’s personalized speed adjustment instructions as the output. This model had been trained for many times to establish a mapping relationship between the operator’s mental state and the speed of the robot. It could adjust the speed of the robot individually according to different mental states, in order to improve the operational quality and system safety.

### Personalized speed adaptation problem formulation

#### Markov decision process

In the personalized regulation loop, the idea of policy gradient reinforcement learning was introduced, and a reinforcement learning model composed of five elements: brain environment, mental state, action indicating the robot speed adjustment command, reward indicating operational quality and CA was built. More specifically, the study found that the mental state of the operator can be changed by the task and the behavior of the robot. For example, when a teleoperated robot performs a difficult task or when the robot makes a mistake, it will trigger the human brain alert (McIntire et al., 2013). When teleoperation tasks are complex and take a long time to perform, brain fatigue can occur due to high mental workload (Warm et al., 2008). When the teleoperation task is too single and simple, it will lead to a decrease in the concentration of the brain (Daly et al., 2017). Therefore, we assumed that the process conforms to a MDP (Chanel et al., 2020). The MDP framework is a convenient choice for planning under uncertainty. This famous stochastic control process is an elegant way to model and solve probabilistic planning problems. Once the possible actions and mental states have been identified, the goal of the problem is defined using a reward function that evaluates the utility of a state-action pair. This makes possible to define the utility of an action sequence as the expected sum of the rewards obtained over time given an initial state. The optimal sequence of actions is the one that maximizes such an expected sum of rewards.

#### Monte Carlo sampling

Unlike reinforcement learning models that know the reward by performing a single-step operation, the PSA model needs to perform a multi-step operation task before getting the reward. At the same time, since the state transition probability and reward function of the model are unknown, the model belongs to the category of multi-step, model-free reinforcement learning. Therefore, we started from Monte Carlo sampling (Speagle, 2020) to design the PSA model. In the case of model-free reinforcement learning, the first problem encountered by the policy iteration algorithm is that the policy cannot be evaluated. This is due to the fact that the model is unknown and cannot do full probability expansion. At this point, the state of the transition and the reward obtained can only be observed by performing the selected action in the environment. Inspired by the *K*-armed bandit, a straightforward alternative to policy evaluation is to “sample” multiple times and then find the average cumulative reward as an approximation of the expected cumulative reward. This is called Monte Carlo reinforcement learning and is also a key point in designing the PSA model (Andrieu et al., 2003).

#### Policy gradient

The policy gradient method is to directly simulate the policy with a neural network. The input of the neural network is the current corresponding state of the agent, and the output is the corresponding action (or action selection probability). The training of the model is actually a process of continuous exploration directly in the policy space to find the optimal policy (neural network parameters). This method works by modeling the policy function and then using gradient descent to update the parameters of the network. It does not have an actual loss function in reinforcement learning, and its purpose is to maximize the expected value of the cumulative reward, so the expected value of the cumulative reward is used as the loss function. The formula is as follows:


(1) $$\nabla J\,\left(\theta \right)=E_{\tau ∼p_{\theta }\,\left(\tau \right)}\,\left[R\,\left(\tau \right)\,\nabla \ln p_{\theta }\,\left(\tau \right)\right]$$

where *R*(τ) represents the reward for sampling trajectory τ. *p*_θ_(τ) refers to the probability of sampling trajectory τ in the case of given neural network parameters θ. For those cases of high-dimensional or continuous state space, after obtaining the value function through the learning based on the value function, when formulating the strategy, it is necessary to compare the value corresponding to various actions. In this way, if the dimension of the action space is high or continuous, it is necessary to compare an action with the maximum value function from it, and this process becomes impractical. However, the policy gradient method can be directly applied to reinforcement learning scenarios in high-dimensional or continuous action spaces. Therefore, this paper chose to design the PSA model based on the policy gradient framework.

Formally, the MDP model of PSA was defined as a tuple (*S*, *A*, *P*, *R*), where:

• *S* is the set of states, this paper represents the set of mental states *s*;
• *A* is the set of actions, this paper represents the set of robot speed adjustment instructions *a*;
• *P* is the transition function, which defines the policy *p* of reaching the state *s*_*i*_ ∈ *S* given that the action *a* ∈ *A* is performed in state *s*_*i*_−_1_ ∈ *S*;
• *R* is the reward function that values any state-action pair, this paper represents the operational quality function;

### Personalized speed adaptation method

**FIGURE 2:**
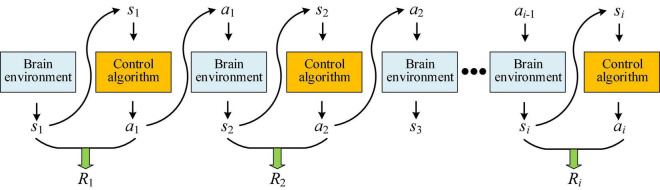
Schematic diagram of Monte Carlo sampling in the PSA model.

According to the framework of policy gradient reinforcement learning, starting from the principle of Monte Carlo sampling, the PSA model was designed. Among them, the multi-step sampling process is shown in Figure 2. Starting from any initial mental state *s*_1_, a certain policy *p* is used for sampling, and the policy is executed for *i* steps and the trajectory τ is obtained. This process can be represented by the following formula (Zhang et al., 2021):


(2) $$\begin{array}{ccc} p_{\theta }\,\left(\tau \right) & = & p\,\left(s_{1}\right)\,p_{\theta }\,\left(c_{1}|s_{1}\right)\,p\,\left(s_{2}|s_{1},c_{1}\right)\,p_{\theta }\,\left(c_{2}|s_{2}\right)\\ & & p\,\left(s_{3}|s_{2},c_{2}\right)\,\cdots \,p_{\theta }\,\left(c_{t}|s_{t}\right)\,p\,\left(s_{t+1}|s_{t},c_{t}\right)\\ & = & p\,\left(s_{1}\right)\,\prod_{t=1}^{T}p_{\theta }\,\left(c_{t}|s_{t}\right)\,p\,\left(s_{t+1}|s_{t},c_{t}\right) \end{array}\}$$

where *s*_*i*_ (*i* = 1,……,*k*) represents the mental state at the moment *i* (hereinafter referred to as the state). *a*_*i*_ (*i* = 1,……,*k*) represents the robot’s speed adjustment action at the moment *i* (hereinafter referred to as the action). *p*_θ_(τ) refers to the probability of sampling trajectory τ in the case of given neural network parameters θ. *p*(*s*_1_) is the probability of the initial state *s*_1_. *p*_θ_(*a*_*i*_|*s*_*i*_) is the probability of taking action *a*_*i*_ given the current state *s*_*i*_. *p*(*s*_*i* + 1_|*s*_*i*_,*a*_*i*_) refers to the probability of returning the next state *s_*i*+_*_1_ based on the conditional probability after taking the current state *s*_*i*_ and action *a*_*i*_. For a certain sampling trajectory τ, the corresponding reward can be obtained. Different rewards can be obtained by optimizing the PSA model. The actions taken by the PSA model and the appearance of a certain state are random. The ultimate goal is to find a policy neural network with the maximum cumulative expected reward ${\bar{R}}_{\theta }$, and according to Formula (1), the objective function is shown as follows:


(3) $${\bar{R}}_{\theta }=\sum_{\tau }R\,\left(\tau \right)\,p_{\theta }\,\left(\tau \right)$$

where *R*(τ) represents the reward for sampling trajectory τ. To calculate the maximum value of the objective function and the corresponding neural network parameter θ, the gradient descent method was adopted. The formula of the gradient $\nabla {\bar{R}}_{\theta }$ of the objective function is shown as follows:


(4) $$\nabla {\bar{R}}_{\theta }\approx \frac{1}{N}\,\sum_{n=1}^{N}R\,\left(\tau^{\left(n\right)}\right)\,\nabla \ln p_{\theta }\,\left(\tau^{\left(n\right)}\right)$$

where *n* is the number of sampling. *N* is the total number of samples. From Formulas (2), (4), the following formula can be obtained:


(5) $$\nabla {\bar{R}}_{\theta }\approx \frac{1}{N}\,\sum_{n=1}^{N}\sum_{i=1}^{k}R\,\left(\tau^{\left(n\right)}\right)\,\nabla \ln p_{\theta }\,\left(a_{i}^{\left(n\right)}|s_{i}^{\left(n\right)}\right)$$

To make the reward value *R*(τ) not affected by the randomness of sampling, a baseline *b* was introduced in this paper. Therefore, the gradient formula is optimized as follows:


(6) $$\begin{array}{c} \nabla {\bar{R}}_{\theta }\approx \frac{1}{N}\,\sum_{n=1}^{N}\sum_{i=1}^{k}\left(R\,\left(\tau^{\left(n\right)}\right)-b\right)\,\nabla \ln p_{\theta }\,\left(a_{i}^{\left(n\right)}|s_{i}^{\left(n\right)}\right)\\ b\approx E\,\left[R\,\left(\tau \right)\right] \end{array}\}$$

**FIGURE 3:**
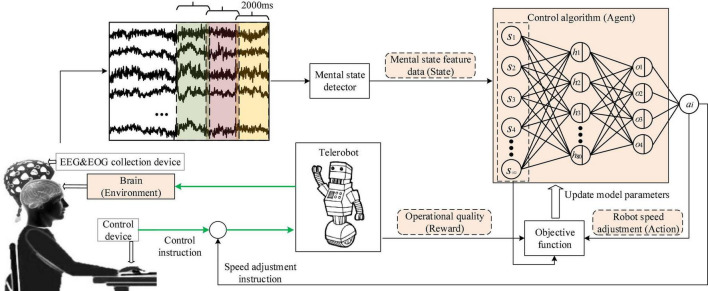
The figure shows the update process of the PSA model.

In Formula (6), the mental state *s*_*i*_ was represented by the feature vector of the collected bioelectric signals, and see Section “Mental state feature extractor” for the detailed formula. The action *a*_*i*_ representing the robot speed regulation command was obtained from the output value of the neural network. The reward *R* represented the operational quality, which was obtained according to the task quality score, and see Section “Personalized speed adaptation model trainer” for the detailed formula. Finally, the gradient descent method was used to update the parameter of the neural network θ. The detailed update process is shown in Figure 3.


(7) $$\theta =\theta +\nabla {\bar{R}}_{\theta }$$

### Personalized speed adaptation model training convergence criteria

The PSA approach is a “human-in-the-loop” reinforcement learning architecture that often exists in a dynamic task environment with no fixed optimal solution, where unpredictable events may require policy changes. In such an environment, conventional convergence rules are not a good criterion for performance evaluation because the agent needs to continuously learn and adapt. For a “human-in-the-loop” reinforcement learning training process, it’s arguably better for the algorithm to learn certain repetitive subsequences of actions (or patterns of actions) and store them in a rule-based fashion. Once an action pattern has been shown to be successful in multiple instances of a task context, it can be applied in similar other task contexts (Wen et al., 2020; van Zoelen et al., 2021). Therefore, we believed that research on reinforcement learning of “human-in-the-loop” should not use conventional convergence criteria as a criterion for whether a model is valid, but should focus on whether a successful action pattern emerges, and its sustainability. Therefore, we updated the PSA model convergence evaluation method, which was evaluated by two indicators: reward and operator’s subjective evaluation. When the fluctuation of the reward is maintained in a relatively small range, and the action level at this time is consistent with the action level expected by the operator’s subjective evaluation. That is to say, the operator is neither strenuous (it will not consume too much mental workload because the difficulty is too high), nor boring (it will not lose the sense of participation or reduce the attention because the task is too easy), we believe that the model training has reached convergence at this time.

## Materials and experiments

### Participants and experimental setup

Six healthy participants took part in this study (the age range was 23−32, 1 female). All participants reported normal or corrected-to-normal vision and had no previous experience with the PSA system. Written informed consent was obtained from each participant before the experiment. The Institutional Review Board of Xi’an Jiaotong University approved the proposed experiment, and all experiments were conducted following the Declaration of Helsinki.

**FIGURE 4:**
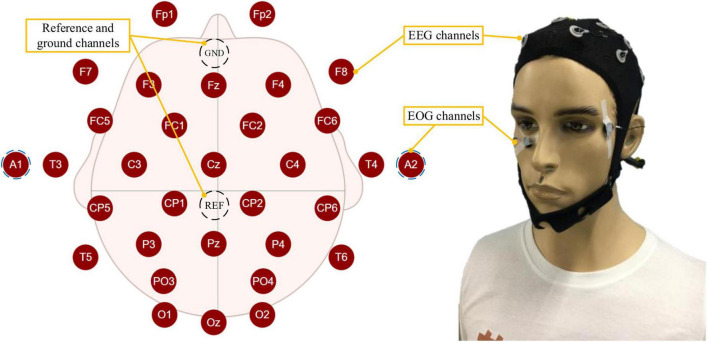
The figure shows the distribution of EEG and EOG electrodes and how the participant wore them.

**FIGURE 5:**
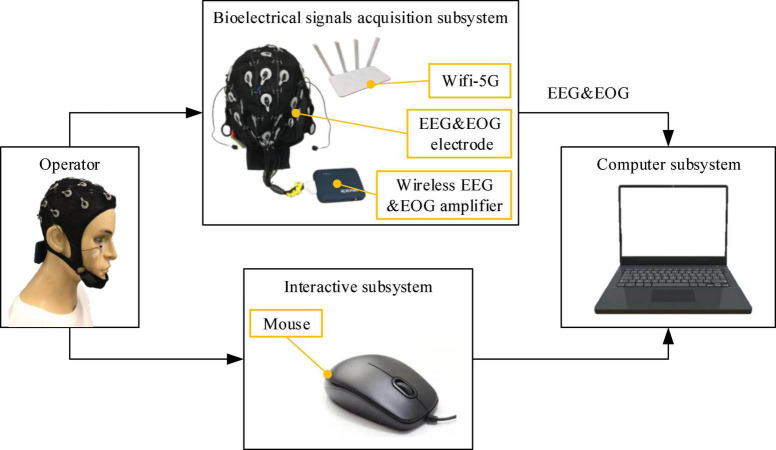
The figure shows the overview of the PSA experimental system.

The PSA experimental system mainly included three subsystems: the bioelectrical signals acquisition subsystem, computer subsystem, and interactive subsystem. The bioelectrical signals acquisition subsystem was mainly responsible for acquiring, amplifying, and transmitting EEG and EOG signals to the computer subsystem. EEG&EOG-W32 model device manufactured by Neuracle Technology Co., Ltd., was used, the sampling frequency was 1000 Hz, and the communication method was WiFi. The device consisted of 30 EEG measuring electrodes, 2 EOG measuring electrodes, 1 reference electrode (REF) and 1 ground electrode (GND). The impedance level of all measuring electrodes were kept below 10 kΩ in each experiment. The electrodes’ distribution conformed to international 10−20 standards (Figure 4). The computer subsystem included two modules, the first one was the PSA module, which was responsible for real-time processing of bioelectrical signals (EEG and EOG), detecting the operator’s mental state, and generating speed adjustment instructions for the robot. This module used MATLAB 2019b and PYTHON3.6. The second was the task simulator module, which was responsible for generating the task environment. This module uses PYTHON3.6. TCP/IP communication was used between the two modules. A microprocessor with Intel (R) Core (TM) i5-5600 CPU was employed in the computer. The interactive subsystem (this refers to the mouse) was used to realize the human-computer interaction function. An overview of the system is illustrated in Figure 5. When the operator controlled the robot through the mouse to perform tasks, the EEG and EOG were collected and transmitted to the computer in real-time. Then, the computer detected the operator’s mental state and sent the speed adjustment instruction adaptively. Specifically, the EEG and EOG cap worn by the operator collected EEG and EOG data in real time while performing the task and sent it to the computer. The computer sequentially performed preprocessing (see section “Multimodal bioelectrical signal preprocessor” for details), feature extraction (see section “Mental state feature extractor”) and PSA model processing (see section “Personalized speed adaptation model trainer”) on these data. Then generated a speed adjustment instruction and sent it to the robot. MATLAB 2019b was used in the data preprocessing and feature extraction steps, and PYTHON 3.6 was used in the PSA model processing steps.

### Experimental task

By analyzing the common characteristics of the telerobot tasks, we abstracted two virtual tasks, namely trajectory tracking and target positioning. Trajectory tracking could simulate tele-operated EOD robots to perform explosive transfer tasks, tele-operated detection robots to perform submarine inspection tasks, and tele-operated AGV robots to perform cargo transfer tasks, etc. Target positioning could simulate the remote-operated weapon system to perform targeting tasks, and the remote-operated aircraft to perform rendezvous and docking tasks with the space station. The specific contents of these two experimental tasks are as follows.

**FIGURE 6:**
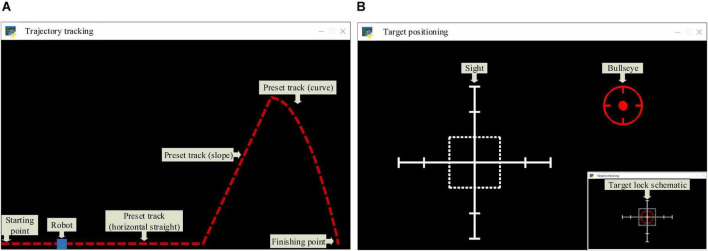
**(A)** The trajectory tracking task requires the operator to control the robot to move from the starting point to the finishing point. The higher the fitting degree of the trajectory and the robot’s preset trajectory, and the shorter the task completion time, the higher the operational quality will be. Besides, three stimulation conditions were set in the experimental task, namely positive mental state stimulation, negative mental state stimulation, and nervous state stimulation. These stimulation conditions were also set in the target positioning task. **(B)** The target positioning task requires the operator to control the sight to track the bullseye. The shorter the time to complete the task, the higher the operational quality will be.

Figure 6A shows the screen of the trajectory tracking task, in which the blue block represents the mobile robot, and the red dotted line represents the preset trajectory. The operator could control the robot to perform the trajectory tracking task through the mouse. Specifically, the operator only needed to move the mouse (without clicking any button), and dragged the robot to move along the preset trajectory. The robot’s position was coupled to the mouse coordinate system. The trajectory of the robot’s movement in each round was recorded by the computer, and the deviation between it and the preset trajectory was calculated. When the robot moved from the starting point to the endpoint, it was regarded as having completed a round of trajectory tracking task. The trajectory deviation and task completion time were recorded to evaluate the trajectory tracking task’s operational quality (the calculation formula is as shown in section “Personalized speed adaptation model trainer”). To increase the diversity of experimental trajectories, three difficulty levels of horizontal straight, slope, and curve were designed; Figure 6B shows the screen of the target positioning task, in which the red center is the bullseye, and the white rectangle is the sight (it can also be considered a robot). The operator could control the sight to perform the target positioning task through the mouse. Specifically, the operator only needed to move the mouse (without clicking any button), and dragged the sight to track the target. When the rectangular frame of the sight fully enclosed the target’s ring and was locked for a while, the target positioning mission was considered successful. The task completion time was recorded to evaluate the operational quality (the calculation formula is as shown in section “Personalized speed adaptation model trainer”) of the target positioning task. The bullseye moved randomly to the next position after each round. The control instructions of the robot were controlled by the operator through the mouse, and the speed adjustment instructions were continuously adjusted by the CA in the PSA model according to the changes in the operator’s mental state. The robot combined the two instructions to perform the tasks. In the task, the operator continuously adjusted the control instruction by observing the robot’s running state. Meanwhile, the CA adjusts the robot’s speed adjustment instruction in real-time by detecting the mental state of the human brain.

### Experimental scenarios and procedures

**FIGURE 7:**
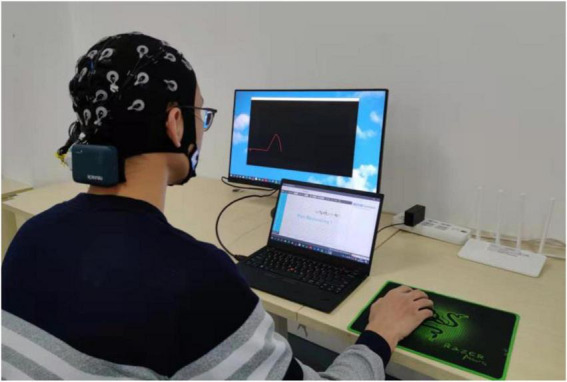
The figure shows the experimental scenario.

**FIGURE 8:**
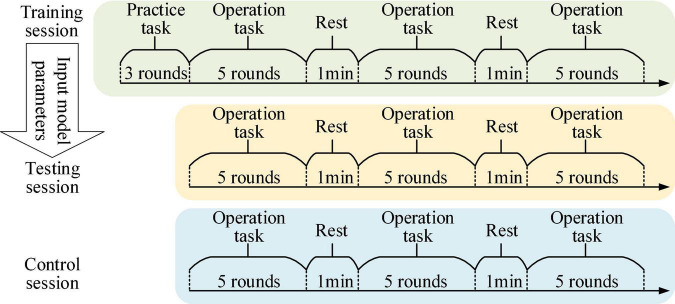
The figure shows the experimental procedure.

In the experimental task of controlling the robot, the participants were asked to sit quietly in front of the computer screen and control the robot or the sight on the screen to perform trajectory tracking or target positioning tasks by mouse, and the experimental scenarios are shown in Figure 7. The experimental tasks were divided into three sessions, namely training session, testing session and control session, and the experimental procedures are shown in Figure 8. Firstly, the training session was used to train PSA model parameters. In the training session, the experiment was performed for 18 rounds. The first 3 rounds were used to practice trajectory tracking and target positioning tasks to prevent different operational proficiency from affecting the experimental results. The last 15 rounds were formal experiments, with 1 min rest time in the middle of every 5 rounds. Secondly, the testing session was used to test the effect of the PSA method. And the trained PSA model parameters were imported into the PSA model in the testing session. In the testing session, the experiment was performed for 15 rounds. Thirdly, the control session was used to provide reference. The conventional method was used in the control session (i.e., this method relies on a warning threshold to trigger an adjustment strategy (Stanney et al., 2009). A warning threshold for whether to enable the adjustment strategy was preset. Then, if the output value of the mental state detector was greater than the warning threshold, the adjustment strategy will be initiated to adjust the speed of the robot; otherwise, the adjustment strategy was not activated. More specifically, when the output value of the mental state detector was higher than the warning threshold, the speed of the robot was reduced. Conversely, when the output value of the mental state detector returned to normal, that is, when it was lower than the warning threshold, the speed of the robot was restored. It is a fixed, non-personalized method of adjustment). In the control session, the experiment was performed for 15 rounds. The effectiveness and superiority of the PSA method were analyzed through the comparison between the testing session and the control session. At the end of each experimental session, the subjects were asked to answer several subjective questionnaire questions. It took about 2 h for each subject to complete the experiment of 3 sessions in total, and the whole experiment lasted 6 days to complete.

Moreover, to increase the diversity of mental states in sampled data, two main measures were adopted. Firstly, three kinds of stimulation conditions were set in the experimental task, namely positive mental state stimulation: a positive text prompt of “Performance is very good” would appear randomly on the screen; negative mental state stimulation: a negative text prompt “Performance is very bad” would appear randomly on the screen; nervous state stimulation: a text prompt of “The Key round” would appear randomly on the display screen, accompanied by the audio prompt of a countdown. Secondly, by setting up the rest time of the experiment, the mental state of fatigue and non-fatigued could be increased. By analyzing the questionnaires of the participants, the settings of these two measures increased the diversity of mental states during the experiment to a certain extent (see section “Changes in mental state during teleoperation experiments” for details).

### Data processing

**FIGURE 9:**
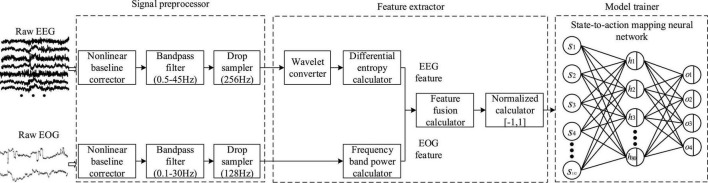
Flow chart of data preprocessing steps.

Data processing mainly includes three steps of multimodal bioelectrical signal (EEG and EOG) preprocessing, mental state feature extraction, and efficient model training. The detailed processing flow is shown in Figure 9.

#### Multimodal bioelectrical signal preprocessor

To feed real-time EEG and EOG into the PSA model, the last 1 min long signal in the computer memory was extracted at each round. A time window of 2000 ms duration was designed for sliding sampling, and 50 sets of raw data were generated. Then, the data was preprocessed, which mainly includes three steps. Firstly, baseline drift in raw EEG and EOG signals was eliminated by the fitted baseline method. The purpose was to eliminate the effects of baseline drift in technical artifacts. The detailed operation method was to fit the trend term by the least squares method, and then subtracted the trend term from the original data. Secondly, the 4th order Butterworth bandpass filter was used to process the two original signals, retaining the EEG of 0.5−45.0 Hz and the EOG of 0.1−30 Hz, respectively. Since EOG also contained mental state information, it was an effective signal in this study, so no artifact processing operation was required for it. Finally, the sampling frequencies of EEG and EOG were down-sampled to 256 Hz and 128 Hz, respectively, thereby reducing the amount of data and improving the calculation speed.

#### Mental state feature extractor

We first introduced the feature extraction method of EEG, which mainly included three steps: (1) Obtained rhythm waves in different frequency bands through wavelet transform. (2) Calculated the four features of sample entropy (*SE*), differential entropy (*DE*), band power (*BP*) and band energy (*BE*) for rhythmic waves of various frequency bands. (3) Calculated the mutual information (*MI*) value between each feature and arousal (or valence) to judge the validity of the feature (it should be noted that the third step was to compare the pros and cons of the four features, which was not required in the actual algorithm running). The following is a detailed introduction. Firstly, the EEG was decomposed and reconstructed using the wavelet basis function of fifth-order vanishing interval Daubechies, and five kinds of rhythmic waves were generated. Their frequency bands are δ (0.5−3 Hz), θ (4−7 Hz), α (8−15 Hz), β (16−31 Hz), and γ (>32 Hz), respectively (Hipp et al., 2011; Dimitrakopoulos et al., 2018). The wavelet transform formula is as follows:


(8) $$x_{j}=A\,C_{j}+\sum_{j=1}^{L}D\,C_{j}$$

where *x*_*j*_ represents EEG of the *j*th frequency band; *L* represents the number of decomposition layers; *AC*_*j*_ represents the approximate component of the *j*th frequency band; *DC*_*j*_ represents the detailed components of different scales of the *j*th frequency band. Secondly, *SE* features, *DE* features, *BP* features, and *BE* features of 5 rhythmic waves from 30 EEG channels were calculated. Studies had found that as the mental state changes, the information complexity of EEG was also changing (García-Martínez et al., 2016; Xu et al., 2019; Ahammed and Ahmed, 2020). For example, when the degree of fatigue increases, the ability of the central nervous system to inhibit brain neural activity may increase, resulting in a decrease in the disordered degree of thinking in the cerebral cortex, thereby reducing the information complexity of EEG (Wang et al., 2011). For another example, Deli and Fry studied positive and negative mental states from the perspective of thermodynamics, and believed that the positive state is in an endothermic cycle (Reversed Carnot cycle), which is a process of absorbing energy from the environment and increasing entropy; In contrast, the negative state is in an exothermic cycle (Carnot cycle), a process that releases energy into the environment and reduces entropy (Deli and Kisvarday, 2020). This also leads to changes in the information complexity of EEG. Therefore, this paper selected representative *SE* and *DE* features for analysis. The *SE* is defined as the negative natural logarithm of the conditional probability that the two subsequences are similar when the pair of subsequences of length *m* are similar after adding one sample point in each order. It can be used to describe the self-similarity and complexity of a sequence (Cuesta-Frau et al., 2017). The lower the *SE* value, the higher the self-similarity of the sequence and the lower the complexity, and the calculation formula of *SE* is as follows (Li et al., 2018):


(9) $$S\,E\,\left(U,v,\eta \right)=-\ln\frac{B^{v+1}\,\left(\eta \right)}{B^{v}\,\left(\eta \right)}$$

where *U* is the sequence length. *v* is the length of continuous subsequence. η is the distance error threshold to judge the similarity of two subsequences. *B^v^*(η) represents the logarithm of the continuous subsequence with length *m* satisfies the similarity condition *r* in the sequence. The parameters *m* and *r* can be determined by cross-validation results on the training set. In this paper, *m* = 2, *r* = 0.2*std, where *std* is the data’s standard deviation. Besides, *DE* could also be used to measure the complexity of temporal sequence signals (García-Martínez et al., 2016). For a fixed-length EEG sequence, the calculation formula of *DE* can be approximated as (Shi et al., 2013):


(10) $$\begin{array}{c} D\,E\,\left(x\right)=-\int_{x}f\,\left(x\right)\,\ln\left(f\,\left(x\right)\right)\,dx\approx \\ -\int_{-\infty }^{\infty }\{\frac{1}{\sqrt{2\,\pi \,\sigma^{2}}}\exp\left[-\frac{{\left(x-\mu \right)}^{2}}{2\,\sigma^{2}}\right]\ln\\ \left(\frac{1}{\sqrt{2\,\pi \,\sigma^{2}}}\exp\left[-\frac{{\left(x-\mu \right)}^{2}}{2\,\sigma^{2}}\right]\right)\}dx=\\ \frac{1}{2}\,\ln\left(2\,\text{π}\,\sigma^{2}\right)+\frac{1}{2}\\ \sigma^{2}=\frac{1}{L}\,\sum_{j=1}^{L}x_{j}^{2} \end{array}\}$$

where *f*(*x*) is the probability density function of time series. μ and σ represent the mean and standard deviation of the Gaussian, respectively. In addition, the study found that the frequency domain characteristics of EEG could also characterize mental state. Therefore, this paper selected two features, *BP* (Liu F. et al., 2021) and *BE* (Matei and Matei, 2021), respectively. Finally, *MI* was used to analyze the amount of mental state information contained in the four EEG features, so as to select the optimal feature. *MI* between these four features (*SE*, *DE*, *BP*, and *BE*) and the valence (or arousal) in dimension theory were calculated separately. In probability theory and information theory, *MI* of two random variables is a measure of the interdependence of variables (Peng et al., 2005). In this paper, the *MI* of *X*_*F*_ and *Y*_*L*_, two random variables representing features and valence (or arousal), respectively, is defined as:


(11) $$M\,I\,\left(X_{\text{F}};Y_{\text{L}}\right)=\sum_{y_{\text{L}}\in Y_{\text{L}}}\sum_{x_{\text{F}}\in X_{\text{F}}}p\,\left(x_{\text{F}},y_{\text{L}}\right)\,\log\left(\frac{p\,\left(x_{\text{F}},y_{\text{L}}\right)}{p\,\left(x_{\text{F}}\right)\,p\,\left(y_{\text{L}}\right)}\right)$$

where *p*(*x*_F_,*y*_L_) is the joint probability distribution function of *X*_*F*_ and *Y*_*L*_. *p*(*x*_F_) and *p*(*y*_L_) are the marginal probability distribution functions of *X*_*F*_ and *Y*_*L*_, respectively. Study found *DE* could effectively characterize mental state (Zheng and Lu, 2015; Zheng et al., 2019; Liu S. et al., 2021) (see section “Analysis of mental state features” for details), hence, it was selected as an EEG feature to represent mental state in the following study. There were 30 EEG channels, and the EEG of each channel was decomposed into 5 rhythmic waves, so the features data of EEG had a total of 150 dimensions.

Then, we introduced the feature extraction method of EOG. It was found that the low-frequency components of EOG increased while the high-frequency components decreased when the arousal level was low (Ma et al., 2014). Therefore, the power ratios (*PR*) of low-frequency components and high-frequency components in the signals were selected as the EOG features representing mental states. The calculation formula is as follows:


(12) $$P\,R=\frac{p\,l\,\left(x\right)}{p\,h\,\left(x\right)}$$

where *pl*(*x*) and *ph*(*x*) represent the power of low-frequency component (0−1.5 Hz) and high-frequency component (1.5−30 Hz), respectively (Magosso et al., 2007; Gao et al., 2012). There were 2 EOG channels, so the features data of EOG had a total of 2 dimensions. In summary, the EEG and EOG features data were combined and normalized to form 152-dimensional characteristic data representing the operator’s mental state.

#### Personalized speed adaptation model trainer

To consider the algorithm accuracy and response speed, a three-layer fully connected state-to-action mapping neural network (SAMNN) was established in the PSA model. The input of the network was mental state *s*_*i*_ and the output was robot speed adjustment instruction *a*_*i*_. There were 152 neurons in the input layer, 80 neurons in the hidden layer, and 4 neurons in the output layer, representing 4 dimensionless speed levels. Tanh activation function (LeCun et al., 2012) was adopted in the hidden layer. The reason was that (1) the network had only one hidden layer, and under the premise of enjoying the advantages of the Tanh activation function, there was no need to worry about the hidden danger of gradient vanishing (Wang et al., 2019). At the same time, (2) the problem of permanent neuron death by using the ReLU activation function was avoided (Nair and Hinton, 2010). Softmax function (Liang et al., 2017) was used for the output layer. The higher the value of the output neuron, the higher the probability of the corresponding action being selected, and vice versa. Then, *s*, *a*, and *R* was input to the objective function [Formula (6)], and the SAMNN parameters were updated according to the adaptive moment estimation method (ADAM) (Kingma and Ba, 2014), Among them, the exponential decay rate for the 1st moment estimates was set to 0.9, and the exponential decay rate for the 2nd moment estimates was set to 0.999. The learning rate was set to 0.001. Each round was an episode, each episode would generate 50 sets of data pairs, and the batch size was 50. The SAMNN parameter was updated once every episode until the model converged.

It is important to note that the reinforcement learning model’s performance is directly affected by the convergence property (Mnih et al., 2015). Therefore, to enhance the convergence performance of the PSA model, shorten the convergence process and improve data utilization, before the online training of the PSA model, the DEAP (Koelstra et al., 2012) dataset was first used to pre-train the SAMNN. The input was feature data representing the mental state, which was from the DEAP dataset. The output was 4 categories representing High valence and High arousal, High valence and Low arousal, Low valence and High arousal, and Low valence and Low arousal, respectively. After 10,000 epoch training, the loss function value was 0.001. It should be noted that before the main model training starts, the pre-trained model parameters need to be imported into the main training model.

In addition, it should be noted that the reward *R* in the PSA model [that is, the *R* in Formula (6)] needed to be calculated according to the specific task, which represented the operational quality. The trajectory tracking reward *R*_*t*_ was evaluated by two indicators of robot trajectory quality and task completion time. The target positioning task reward *R*_*p*_ was evaluated by the indicator of task completion time. The calculation formula is as follows:


(13) $$\begin{array}{c} R_{\text{t}}=\frac{1}{\sum_{m=1}^{M}\left|Y_{m}-O_{m}\right|}+\frac{g}{t}\\ R_{\text{p}}=\frac{g}{t}\;\mathrm{if}\,t\geq T \end{array}\}$$

where *Y* represents the trajectory of the robot. *O* represents the target trajectory. *t* is the time to complete each round. *g* is the time gain coefficient. *M* represents the total number of steps of the whole trajectory, and *T* represents the time threshold of the sight continuously aiming at the bullseye.

## Results

### Analysis of mental state features

**FIGURE 10:**
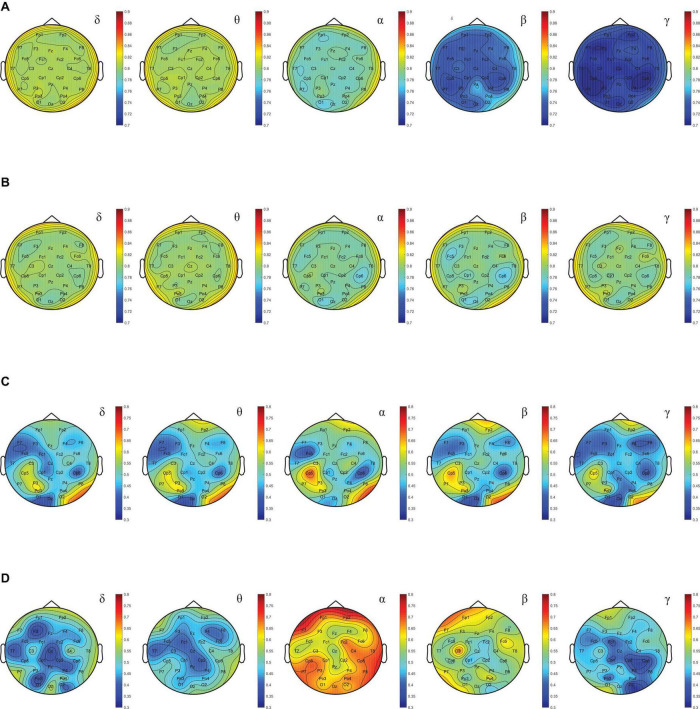
**(A)** Brain topography based on *MI* between *SE* and valence. **(B)** Brain topography based on *MI* between *DE* and valence. **(C)** Brain topography based on *MI* between *BP* and valence. **(D)** Brain topography based on *MI* between *BE* and valence.

In order to find EEG features that can stably and effectively represent mental state, we used the DEAP dataset to calculate the average of the four EEG features (*SE*, *DE*, *BP*, and *BE*) described above, and the *MI* between these four averages and valence. The higher the *MI*, the more mental state information was contained in the feature data, and vice versa. For a more intuitive display, for each feature, the *MI* of each rhythmic wave in 30 EEG channels was calculated separately, and the brain topographic map was drawn according to the *MI* value. Figure 10A shows the *MI* brain topography between *SE* and valence for the five rhythmic waves. By contrast, it was found that *MI* decreased with increasing rhythmic wave frequency. Figure 10B shows the *MI* brain topography between *DE* and valence for the five rhythmic waves. By comparison, it was found that the *MI* not only did not show an obvious decreasing trend, but also all channels remained at a relatively high level (the average was 0.85), which indicated that the *DE* features contained more information of mental state and had a high stability. Figures 10C,D show *MI* brain topography between *BP*, *BE*, and valence for the five rhythmic waves, respectively. Both *MI* values showed a low level (average values were 0.4, 0.3, respectively), and the volatility between each rhythm wave was large, and the volatility between each channel was also large. Comprehensive comparison found that the *MI* value of the *DE* feature data was the highest and the stability was strong. At the same time, the study found that the *MI* between these four features and arousal also had the same regularity. To sum up, the *DE* feature contained the most mental state information, and the PSA method achieved the expected effect in the experiment, which also proved the reliability of this phenomenon. Therefore, *DE* features were selected as EEG features to represent mental states.

### Changes in mental state during teleoperation experiments

In order to analyze the change characteristics of the subjects’ mental state during the experiment, we integrated the data recorded by the subjective evaluation scale to generate Table 1. The fatigue level of all subjects changed during the experiment. All subjects except subject 2 experienced changes in their stress level. Except for subjects 4 and 6, the positive and negative mental states of the other subjects changed. It showed that by setting the task difficulty and auxiliary stimulation conditions, the subjects could be induced to induce different mental state. Comprehensive analysis found that subject 1, subject 3, and subject 5 had changes in the degree of fatigue, stress and positive/negative states. Compared with other subjects, these three subjects were more likely to have mental state fluctuations, which was also a manifestation of individual differences.

**TABLE 1:** Subjective evaluation results of mental state during the experiment.

	Has fatigue level changed?	Has the stress level changed?	Have positive and negative mental states changed?
Subject1	Yes	Yes	Yes
Subject2	Yes	No	Yes
Subject3	Yes	Yes	Yes
Subject4	Yes	Yes	No
Subject5	Yes	Yes	Yes
Subject6	Yes	Yes	No

**FIGURE 11:**
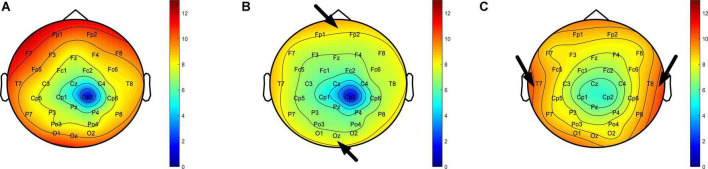
**(A)** Brain topography in normal state. **(B)** Brain topography in fatigue state. **(C)** Brain topography during stress state.

Taking subject 3 to perform the trajectory tracking task as an example, we recorded his EEG in three states of normal, fatigue and stress during the experiment, and calculated the *DE* feature of the EEG in 30 channels, and then drawn the brain topography according to *DE* value. Firstly, by comparing the brain topographic maps of the fatigue (Figure 11B) and normal state (Figure 11A), it was found that the *DE* value decreased when the subject moved from the normal state to the fatigue state, indicating that the complexity of the EEG was reduced. This is in line with previous studies, where one explanation is that fatigue induces the inhibition of cerebral cortical activity by the central nervous system, resulting in a reduced degree of disorder in the EEG of the cerebral cortex (Liu et al., 2010; Wang et al., 2011; Xu et al., 2019). In addition, in the fatigue state, the *DE* values of the occipital lobe, part of the parietal lobe and the prefrontal lobe region decreased to a greater extent (the regions indicated by the arrows in Figure 11B), indicating that these regions are closely related to the processing of the fatigue state. And this phenomenon had also been confirmed in previous studies (Chuang et al., 2018; Ma et al., 2019; Liu et al., 2020). Secondly, by comparing the brain topographic maps in the stress state (Figure 11C) and the normal state (Figure 11A), it was found that the *DE* values in both temporal lobes increased in the stress state (the regions indicated by the arrows in Figure 11C). This phenomenon is consistent with previous studies, and one explanation is that the temporal lobe is involved in the processing of stress states, which are closely related to stress states (Hosseini and Naghibi-Sistani, 2011; Lucassen et al., 2014; Choi et al., 2015; Katmah et al., 2021).

**FIGURE 12:**
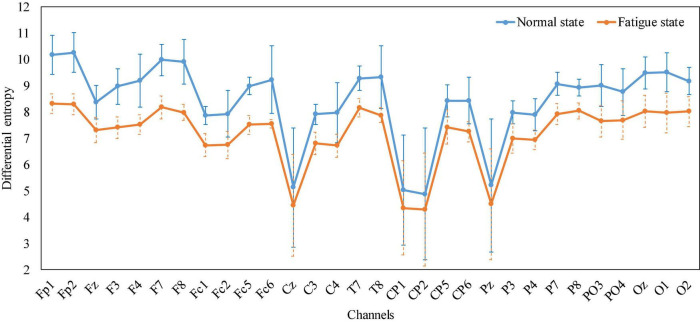
Line graph of the average *DE* characteristics of all subjects in normal and fatigue states, respectively.

Without loss of generality, the average properties of all subjects’ mental states while performing the trajectory tracking task were analyzed. Taking the fatigue state as an example, the reason is that in the experiment, each subject reported that the fatigue state appeared. We recorded the EEG when all subjects reported fatigue during the experiment, calculated the *DE* characteristics of the EEG in 30 channels separately, and plotted a line graph based on their mean and standard deviation. Figure 12 shows that, relative to the normal state, the *DE* value of the fatigue state is reduced, especially in the occipital region, part of the parietal region and the prefrontal region, which is also consistent with the phenomenon in Figure 11B. The generality of EEG features in the fatigue state was demonstrated. To sum up, by analyzing the EEG characteristics of the subjects when they appeared in various mental states during the experiment, and it was found that the same phenomenon existed in previous studies, which proved that the subjects would produce different mental states during the experiment.

### Feasibility analysis of personalized speed adaptation

**FIGURE 13:**
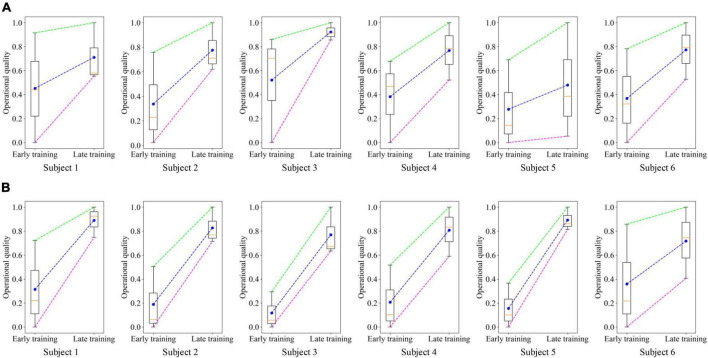
Boxplots were drawn based on the operational quality of all subjects in the early (the first 3 rounds) and late (the last 3 rounds) stages of PSA model training. The dots in the figure represent the mean value, and the horizontal line represents the median. **(A)** Trajectory tracking task. **(B)** Target positioning task.

**FIGURE 14:**
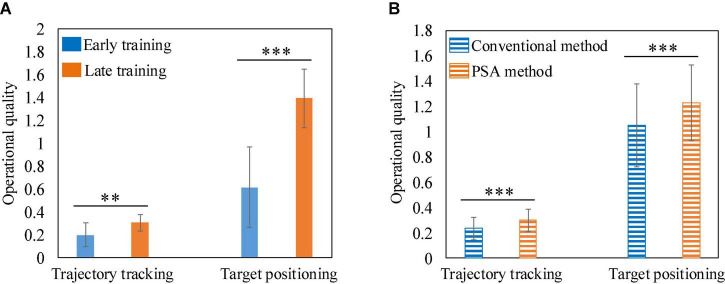
**(A)** The comparison histogram of operational quality between early and late training. **(B)** The comparison histogram of operational quality between the conventional method and the PSA method. **Represents *p* < 0.01, and *** represents *p* < 0.001.

To demonstrate the feasibility of the PSA method, we recorded the operational quality of each subject at the early (the first 3 rounds) and late (the last 3 rounds) training stages, normalized the data, and plotted it into boxplots (Figure 13). In the two experimental tasks, the operational quality of each subject in the late training stage was significantly improved compared with the early training stage. A total of 36 data pairs were formed by recording the operational quality data of all subjects at the early and late stages of training. Statistical analysis using two-sample *T*-test found that there was a significant difference in the operational quality between the two periods, as shown in Figure 14A. It showed that in the training process of the PSA model, CA gradually learned the mapping relationship between each subject’s mental state and the robot’s speed adjustment instructions, and could adjust the robot’s speed in real time according to the mental state, thereby improving the operational quality. In addition, in the trajectory tracking task, the interquartile range (IQR) value of each subject (except subject 5) at the later stage of training was lower than that at the early stage of training. In the target positioning task, the IQR value of each subject (except subject 3) was lower in the late training period compared to the early training period. At the same time, Figure 14A shows that in both experimental tasks, the standard deviation of the operational quality data in the later stage of training is smaller than that in the early stage of training. Both phenomena illustrated that after training, the PSA model gradually converges and the stability gradually improves. Taken together, the feasibility of the PSA method was demonstrated by these phenomena.

### Superiority analysis of personalized speed adaptation

In order to demonstrate the superiority of the PSA method, we choose the current conventional adjustment method based on mental state and the PSA method for comparison, that is, the comparison between the control session and testing session experiments. In the control and test groups of each experimental task, the operational quality data of 6 subjects who performed 15 rounds of the task were recorded, forming a total of 90 data pairs. The mean and standard deviation of these data were calculated, respectively, and then a contrast histogram was drawn. Figure 14B shows that the average operational quality of the PSA method is better than that of the conventional method in both experimental tasks. And the statistical analysis by the two-sample *T*-test found that there was a significant difference in the mean between the two. In conclusion, the validation of 6 subjects in two independent experimental tasks showed that the PSA method was superior to conventional mental state-based adjustment methods. Furthermore, since the feasibility and superiority of the PSA method could be verified on two experimental tasks with different levels of difficulty and task modes, it was proved that the PSA method has good universality.

### Personalized analysis of personalized speed adaptation

**FIGURE 15:**
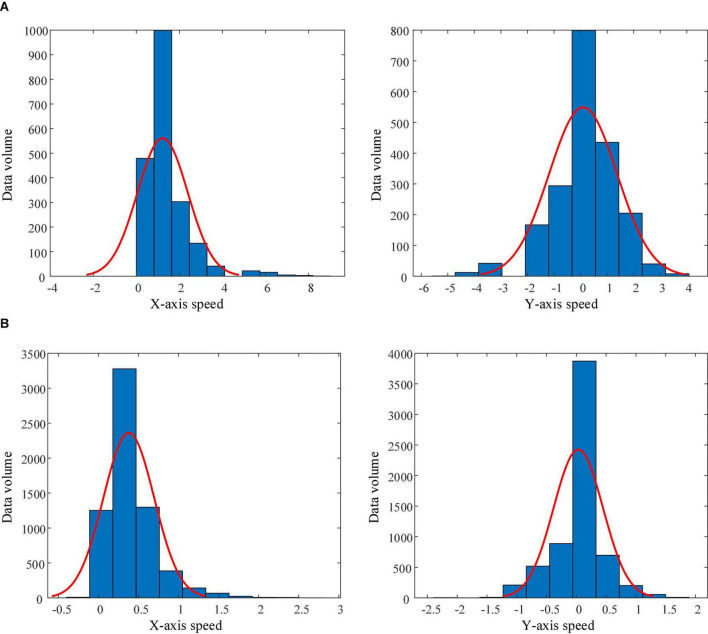
Histograms and fitting curves of the speed distributions of the robots controlled by aggressive style and conservative style subjects. **(A)** Aggressive style subject. **(B)** Conservative style subject.

Through the analysis of the robot speed adjustment instructions (actions) output by the trained PSA model for each subject, it was found that the PSA method had been individually adjusted according to the mental state of different subjects. Referring to previous studies on the classification of operators (or drivers) style (Sadrpour et al., 2014; Gilman et al., 2015; Wang et al., 2020), we divided the subjects into aggressive and conservative types according to the speed of the robot controlled. Subject 1 and 5 belonged to the aggressive style, and the other 4 subjects belonged to the conservative style. Taking the trajectory tracking task as an example, we plotted the distribution histogram and fitting curve of the robot speed data controlled by the aggressive and conservative subjects during the task (Figure 15). Figure 15A shows that the robot controlled by the aggressive subjects has a wide range of *X*-axis velocity distribution (0∼9.0), the mean value of the velocity distribution is 1.22, and the standard deviation is 1.17; and the *Y*-axis velocity distribution range is also wide (−5.0∼4.0), the mean value of the velocity distribution is 0.09, and the standard deviation is 1.28. It showed that the aggressive subjects paid more attention to the sense of control when manipulating the robot, and there would be more rapid acceleration and deceleration. However, Figure 15B shows that the robot controlled by conservative subjects has a narrow range of *X*-axis velocity distribution (−0.3∼2.7), the mean value of velocity distribution is 0.38, and the standard deviation is 0.32; and the *Y*-axis velocity distribution range is also narrow (−2.1∼1.8), with a mean value of 0.03 and a standard deviation of 0.41. It showed that conservative subjects paid more attention to the stability when manipulating the robot and avoid rapid acceleration and deceleration. Through this phenomenon, it was fully proved that the PSA method can perceive the differences between operators and make individual adjustments to the robot speed according to the mental state.

### Visual analysis of personalized speed adaptation

**FIGURE 16:**
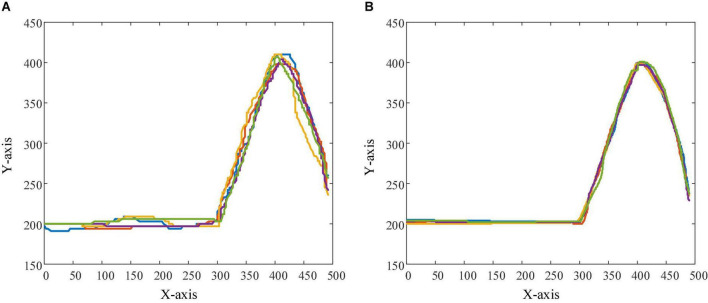
The figure shows the trajectories of the mobile robot in the trajectory tracking task. **(A)** Robot trajectories in the control session. **(B)** Robot trajectories in the testing session.

**FIGURE 17:**
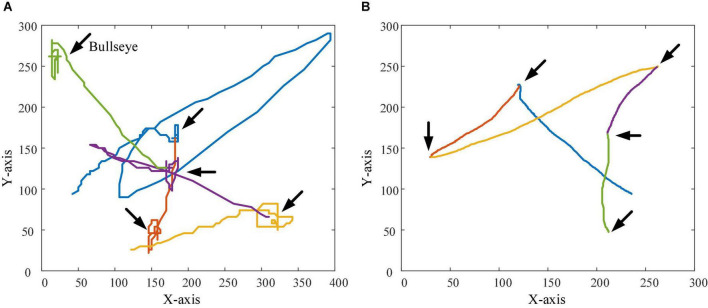
The figure shows the trajectories of the sight in the target positioning task. The position indicated by the arrow in the figure is the bullseye position. **(A)** The trajectories of the sight in the control session. **(B)** The trajectories of the sights in the testing session.

In order to observe the effectiveness and superiority of the PSA method in the two experimental tasks more intuitively, the trajectories of subject 1 when controlling the robot (and sight) to perform the task in the testing session and the control session were recorded 5 times, respectively, as shown in Figures 16, 17. In the trajectory tracking task, it can be seen from the relatively fluctuating trajectories of the control session that the mobile robot frequently loses the trajectory target (Figure 16A), so the operator needs to constantly adjust the control instructions. Not only does this lead to longer time spent on the entire task, but it also increases mental workload and negative emotions. For the testing session using the PSA method, the fit of the mobile robot’s motion trajectory and the target trajectory is better than that of the control session, and the phenomenon of missing targets is reduced (Figure 16B). From the relatively smooth motion trajectories, it could be seen that the operator could control the mobile robot’s travel trajectory compliantly, and did not need to adjust the control commands frequently, and the operation was more accurate and efficient. This phenomenon also exists in the target positioning task, especially when the sight is getting closer and closer to the bullseye, the trajectories of the control session shows that the sighting frequently loses the position of the bullseye. The trajectories at the 5 bullseye positions are like a mess of ropes, as indicated by the arrows in Figure 17A. This not only results in longer time spent on the entire task, but also increases the operator’s mental workload and negative emotions. For the testing session using the PSA method, the trajectories show that the sight can quickly locate and lock the bullseye, no matter whether it is close to the trajectories of the bullseye stage, or the trajectories of the locking stage of the bullseye has been improved (Figure 17B). This showed that the operator could accurately control the sight, and did not need to adjust the control commands frequently for repeated positioning, and the operation was more accurate and efficient.

Without loss of generality, we listed the performance of all subjects in the test and control sessions. In the trajectory tracking task, Table 2 shows that the operational quality of each subject (except subject 6) under the PSA method is better than that of the conventional method, and in the target positioning task, the operational quality of each subject under the PSA method is also better than that of the conventional method. Moreover, in both experimental tasks, the mean operational quality of the PSA method was also superior to that of the conventional method, thus proving the general applicability of the PSA method.

**TABLE 2:** Operational quality of all subjects.

	Trajectory tracking task		Target positioning task	
		
	Operational quality under conventional method	Operational quality under PSA method	Operational quality under conventional method	Operational quality under PSA method
Subject1	0.18	**0.26**	1.14	**1.41**
Subject2	0.23	**0.31**	1.19	**1.23**
Subject3	0.18	**0.29**	0.72	**1.05**
Subject4	0.31	**0.32**	0.98	**1.20**
Subject5	0.25	**0.36**	1.14	**1.26**
Subject6	**0.25**	0.24	1.13	**1.21**
Average	0.23 ± 0.05	0.30 ± 0.04	1.03 ± 0.18	**1.23 ± 0.10**

Bold text indicates better values.

## Discussion

This paper aims to study the feasibility of the PSA method based on mental state for teleoperated robots. The PSA model based on policy gradient reinforcement learning was established, and related algorithms were developed and verified by experiments on real subjects instead of simulation models. The following sections focus on personalization, rapid reinforcement learning, and the scalability of the method in application areas. Then, the existing limitations and future work are prospected.

### Personalization

Due to the huge number of teleoperators involved in various fields of operation, different operators have obvious differences in age, personality, psychological state, and proficiency, as well as the inherent complexity of the operator’s mental state and behavior. As a result, the differential representation of the operating habits and qualities of different operators has become a difficult problem. In the adjustment strategy design of the conventional teleoperated robot system, the method of adapting to the operator’s behavior through parameter calibration is difficult to meet the individual needs of a large number of operators. Different from the conventional subjective (i.e., biased by developer experience) and fixed adjustment methods, the PSA method is objective and flexible (Sogaard et al., 2019; Tang et al., 2019; Wen et al., 2020). The first reason is that the PSA method is designed based on a reinforcement learning architecture, which can obtain feedback rewarded with operational quality through the interaction between the CA and the brain environment, to capture the mapping between various mental states and robot speed regulation commands in real time. Then, the mapping between the various mental states and the robot’s speed-regulating commands is captured in real time. In this way, a personalized “human-in-the-loop” teleoperated robot system model is dynamically established. It can better solve the problems that are difficult to overcome by conventional methods. For example, Figure 15 shows that under the adjustment of the PSA method, the distribution of the speed (action) data of the robot controlled by the subjects has changed, indicating that the method has established a personalized adjustment strategy for each subject. Not only that, as the operating time increases, the operator’s cognitive level and operation skills of the system are also continuously improved, and the operation habits are also changed. The second reason is that the CA (agent) in the PSA model collects more and more personalized, comprehensive information about the operator. Based on this data, model parameters are continuously optimized and adjusted, allowing each teleoperated robotic system to evolve toward a personalized direction. Therefore, the teleoperated robot system equipped with the PSA method also has the ability of lifelong learning and continuous evolution.

### Rapid reinforcement learning

Personalized speed adaptation is a reinforcement learning method based on “human-in-the-loop,” which includes a typical online interactive learning process and has unique advantages, such as personalization, evolution, and better dynamic adaptability. However, due to the difficulty in ensuring the convergence and training efficiency of the “human-in-the-loop” reinforcement learning model, most of the current research is mostly carried out on simulated human models. For example, Wu et al. (2022) established a robot knee tracking control method based on “human-in-the-loop” reinforcement learning, which was verified in a so-called realistic human-robot system simulator. To the best of our knowledge, there is little research and experimental validation on human models, and even less in the field of teleoperated robotics. One reason is that the human brain is an element in the model, which increases the model’s complexity and uncertainty. As Xuesen Qian pointed out, the living system, especially people with advanced psychological activities, is an open and complex giant system. In this case, the control actions are essentially infinitely flexible (Sheridan, 2011). Another reason is that such methods have high data acquisition costs and labor-intensive problems. In this paper, the following efforts are made to solve the problem of rapid learning of the “human-in-the-loop” reinforcement learning model: In terms of algorithms, (1) by setting pre-training, the existing data sets were used to accelerate the learning process of online tasks. (2) By reducing the dimensional space of state and action, the network structure of SAMNN was simplified and the difficulty of network training was reduced. In terms of experimental paradigm, (1) the diversity of samples was increased by setting stimulation conditions in the experiment. (2) A reasonable number of experimental rounds was designed after many attempts. The study found that too many experiment times will reduce the experimental experience of the subjects, but too few experiment times often cannot achieve convergence. It has to be admitted that there are some subjects who can achieve convergence after several attempts. To sum up, after various efforts, the PSA model achieved rapid learning with the participation of real people. However, this work still needs to be further improved, and the next step will be to explore the following aspects: (1) Introduce a meta-learning strategy, meta-learning is to use past knowledge and experience to guide the learning of new tasks, so that the network has the ability to learn to learn, and it is one of the commonly used methods to solve the few-shot learning problem. Theoretically, the meta-reinforcement learning algorithm can enable the agent to learn new skills from a small amount of experience. Although there are some drawbacks, it is a way to try (Rakelly et al., 2019). (2) Introduce the bootstrapped policy gradient rapid reinforcement learning strategy. The bootstrapped policy gradient method can introduce prior knowledge into the policy gradient to improve sample efficiency. Its core idea is to update the sum probability of a series of related actions in the gradient estimation sample, rather than the sum probability of a single action (Zhang and Goh, 2021; Zhang et al., 2021).

### Implications of the results

In this paper, only the speed parameter of the robot was selected as the adjustment variable for research, and exciting results were obtained. The research shows that on the basis of the PSA method, other parameters can be selected as adjustment variables according to the characteristics of the specific teleoperating system and the robot. Furthermore, the PSA method can realize not only the adjustment of a single parameter, but also the coupled adjustment of multiple parameters. It should be noted that when a single parameter is adjusted, the complexity of the teleoperated robot system is relatively easy to determine, but when multiple parameters are adjusted in coupling, the complexity of the system will increase dramatically due to the coupling relationship between the parameters.

The PSA method has been verified in the two tasks of trajectory tracking and target positioning. These two tasks are abstracted according to the common characteristics of the tasks of the teleoperated robot system, and do not depend on any specific teleoperated robot system. Therefore, the PSA method can be well extended to a variety of teleoperated robotic systems. For example, in the field of manipulating special operation robots, such as remote-operated fire-fighting robots, maintenance robots, surgical robots, and space station maintenance robots (Bucolo et al., 2022). The set adjustment parameters can be the response time of the robot system, the speed and acceleration of the moving parts of the robot, and the coupling parameters between them, etc. At the same time, it also has application prospects in other fields, such as information matching and recommendation fields, such as education and training, web page information recommendation (Lan and Baraniuk, 2016; Mizgajski and Morzy, 2019). The set adjustment parameter can be the difficulty level of the task (or event).

### Limitations and future work

The core of the PSA method based on the reinforcement learning framework is to learn the mapping from the mental state (state) to the robot speed regulation instruction (action), which is an end-to-end learning method. Its advantage is that it has the learning ability of non-linear mapping, and it does not need to abstract the rules of the teleoperated robot process, nor to establish a mathematical model between EEG indicators and behavioral performance, thus avoiding the cumulative bias introduced by oversimplifying the study subjects. However, it has to be admitted that its learning process is “black box” and has poor interpretability (special research in this area can be carried out in the next step). Even so, this still cannot hide the unique advantages of the PSA method.

The mental states in this study can be defined in many ways, with different meanings in different disciplines. This paper draws on the definition method of dimension theory in psychology, because it evaluates mental state on a continuous dimension through indicators such as design valence and arousal. The evaluation criteria can fully take into account the characteristics of the diversity of mental states. In the future, the mental state detector in the PSA method can be specially designed and improved for specific fields. A specific, single mental state can be selected for more targeted research. For example, to study the influence of mental fatigue on the teleoperation robot system, it is only necessary to replace the feature extraction method in the mental state detector. The PSA method can realize the function of special customization and rapid transplantation, so as to adapt to the teleoperated robot system with special requirements.

In related fields such as teleoperated robots and car driving, the driving styles of operators (or drivers) are mostly divided into conservative and aggressive types according to driving habits and the speed of the robot (or car) being driven (Sadrpour et al., 2014; Gilman et al., 2015; Wang et al., 2020). Referring to research findings in related fields, we divided the subjects into aggressive and conservative types according to the speed of the robot controlled. However, it has to be admitted that there are still some limitations. Firstly, the existing research on driving style has not formed a unified conceptual framework, and there is no general scheme for the classification of driving style (Sagberg et al., 2015). Secondly, in the field of teleoperation, the theoretical basis for the classification of driving styles and objective group differentiation methods need further exploration.

In this paper, there was no special method for removing electromyogenic artifacts in signal processing, but filtering (retaining the maximum frequency of EEG to 45 Hz) was used to reduce the influence of electromyogenic artifacts as much as possible (Hipp and Siegel, 2013). At the same time, it can ensure the efficient running of the algorithm to meet the requirements of online training for the speed of the algorithm. However, it has to be admitted that when the frequency band of EEG is less than 45 Hz, it is still unable to remove all electromyogenic artifacts. After balancing the advantages and disadvantages of various aspects, we designed this processing method suitable for the working conditions of this paper. In the future, we need to deepen research from two perspectives: (1) Develop a more efficient electromyogenic artifact removal method that is suitable for our online training. (2) Further quantitatively evaluate the advantages and disadvantages between the developed electromyogenic artifact removal method and the low-frequency EEG preservation method, such as the degree of electromyogenic artifact elimination, the degree of effective signal mis injury, and the algorithm running rate.

This paper selects 6 subjects to participate in the online experiment, the main reasons are as follows: Different from other methods that need to verify cross-individual characteristics, they use a large number of subjects to verify their cross-individual accuracy, robustness and stability, however, the focus of the PSA method is on the study of individualized adjustment for the mental state of different operators, not only does not involve cross-individual verification, but instead focuses on the differences between individuals. In the experimental validation involving 6 subjects, it has been observed that the PSA method has carried out personalized regulation for aggressive style and conservative style subjects. And at the same time, it also avoids the drawback that the overall sample is too large to blur the characteristics of the data. In addition, the effect of the PSA method in a single subject is robust and has significant differences. Therefore, this paper used 6 subjects for experimental verification (Fischer and Whitney, 2014).

## Conclusion

Aiming at the problem that the poor mental state of the teleoperator causes the quality of the operation to decline, or even dangerous, the PSA model based on policy gradient reinforcement learning was established in this paper. This model had a dual-loop human-computer information interaction mechanism, which could give full play to the advantages of humans and computers. At the same time, the PSA algorithm was developed, which could extract the *DE* feature of EEG and the *PR* feature of EOG, and performed feature-level fusion to obtain a data matrix that effectively characterizes the mental state. In addition, by fusing the perceptron based on artificial neural network and the decision maker based on reinforcement learning, the function of individually adjusting the speed of the robot according to the mental state of different users was realized. Experiments were carried out on 6 real subjects instead of simulation models. The results showed that the method could accurately perceive the mental state of the operator when performing the task, and the speed of the robot was individually adjusted according to the mental state of different operators, which effectively improved the operational quality and realized the efficient and safe execution of teleoperation tasks. Aiming at the problem of performance degradation of teleoperated robotic systems caused by human factors, this research result may inspire a new control framework. Compared with the conventional methods based on user behavior model mining, a series of methods based on this framework have better personalization and dynamic adaptability.

## Data availability statement

The raw data supporting the conclusions of this article will be made available by the authors, without undue reservation.

## Ethics statement

The studies involving human participants were reviewed and approved by the Institutional Review Board of Xi’an Jiaotong University. The patients/participants provided their written informed consent to participate in this study. Written informed consent was obtained from the individual(s) for the publication of any potentially identifiable images or data included in this article.

## Author contributions

TZ proposed and did the research and wrote the manuscript. XZ proposed the research idea, supervised the work, and revised the manuscript. ZL organized and carried out the experiments. YiZ assisted in processing experimental data. ZJ assisted in collecting experimental data. YnZ revised the manuscript. All authors contributed to the article and approved the submitted version.
